# *Leymus chinensis* resists degraded soil stress by modulating root exudate components to attract beneficial microorganisms

**DOI:** 10.3389/fmicb.2022.951838

**Published:** 2022-12-09

**Authors:** Yulong Lin, Linlin Mei, Qianhao Wei, Bing Li, Pan Zhang, Shixuan Sun, Guowen Cui

**Affiliations:** School of Animal Science and Technology, Northeast Agricultural University, Harbin, China

**Keywords:** forage grass, stress response, root exudates, microbial community, metabolomics

## Abstract

Phytoremediation is an effective means to improve degraded soil nutrients and soil structure. Here, we investigated the remediation effects of *Leymus chinensis* on the physicochemical properties and structure of degraded soil after 3 years of cultivation and explored the bacterial and fungal drivers in root exudates by metabolomics and high-throughput sequencing. The results showed that root exudates increased soil organic matter (SOM), total nitrogen (TN), total phosphorus (TP) and soil aggregates, and organic acids in root exudates reduced pH and activated insoluble nutrients into forms that are available to plants, such as available nitrogen (NH_4_^+^-N), nitrate nitrogen (NO_3_^−^-N), and available phosphorus (AP). The cultivation of *L. chinensis* restored the diversity and richness of soil microorganisms and recruited potential beneficial bacteria and fungi to resist degraded soil stress, and *L. chinensis* also regulated the abundances of organic acids, amino acids and fatty acids in root exudates to remediate degraded soils. Spearman correlation analysis indicated that glutaric acid, 3-hydroxybutyric acid and 4-methylcatechol in root exudates attracted *Haliangium*, *Nitrospira* and *Mortierella* to the rhizosphere and dispersed the relative abundance of the harmful microorganisms *Fusicolla* and *Fusarium*. Our results demonstrate that *L. chinensis* enhances soil fertility, improves soil structure, promotes microbial diversity and abundance, and recruits potentially beneficial microorganisms by modulating root exudate components.

## Introduction

Healthy soil plays a key role in human survival. Unfavorable environmental factors and inappropriate human utilization (including intensive agriculture, fertilizer application, and urban development) lead to the destruction of soil ecosystems and a sharp decline in soil quality (Xie et al., 2014) through erosion, soil organic matter (SOM) loss, and soil compaction, even altering the community structure and diversity of soil microorganisms. These changes have affected the biological and economic productivity of soil microorganisms, representing a worldwide disaster (Koch et al., 2013; Li et al., 2020b). At present, one-third of the world’s available agricultural soil suffers from varying degrees of degradation (FAO and ITPS, 2015). More than 466 million hectares of land in China are degraded (Jie et al., 2002). Degraded soil restoration and sustainability are imperative and emergent. Phytoremediation has become a prevalent strategy for degraded soil remediation due to its environmental friendliness and sustainable advantages (Liu et al., 2018; Ali et al., 2020; Wei et al., 2021; Bhanse et al., 2022; Bhat et al., 2022). Roots cope with a variety of biotic and abiotic stresses at the root-soil interface by secreting various organic and inorganic chemicals that play an important role in resisting environmental stress and the process of phytoremediation (Bais et al., 2006).

Root exudates are complex in chemical composition and typically comprise sugars, amino acids, organic acids, and other secondary metabolites, and their composition and release rates vary with plant species and environmental factors (Rohrbacher and St-Arnaud, 2016; Hu et al., 2018; Schmidt et al., 2019). Root exudation processes are regulated by a series of biological and abiotic factors, such as plant species, soil nutrients, soil structure, temperature and water (Yin et al., 2013; Wang et al., 2019). Roots are also frequently disturbed by various soil environments, and they may also regulate the composition and content of root exudates to mitigate these disturbances (Badri and Vivanco, 2009). Root exudates directly or indirectly affect soil nutrient content, soil physicochemical properties and soil structure (Lin et al., 2018). The components of root exudates vary across plant species, and the release rate is also significantly affected by light conditions and temperature (Zhai et al., 2013). Shen et al. (2020) found that degraded soil nutrient status in grasslands increases root exudation rates. Hamilton et al. (2008) found that defoliation in a natural grassland community stimulated a 1.5-fold increase in root C exudates, which subsequently benefited deciduous plants by enhancing rhizosphere N mineralization. Therefore, root exudates are essential for resisting degraded soil environments and improving soil chemical and biological properties.

Plant root exudates strongly affect the distribution and activity of microorganisms in the soil environment. Thus, microbial activities are generally enhanced in the rhizosphere compared with the non-rhizosphere, and the rhizosphere serves as one of the most dynamic niches (Zeng et al., 2021; Debray et al., 2022). The composition of these rhizosphere microbial communities varies depending on root exudates, soil factors, and plant types (Lima et al., 2015; Lu et al., 2018). Roots also attract, prevent or kill rhizosphere microbes by root exudates and shape specific rhizosphere microbial communities (Qu et al., 2021; Xue et al., 2022). For example, leguminous plants exude specific signal molecules such as flavonoids, to attract nitrogen-fixing bacteria, and the malic acid and citric acid in tomato root exudates have been demonstrated to attract *Pseudomonas fluorescens* strains (Weert et al., 2002; Broughton et al., 2003). Recently, more evidence has shown that these microbial communities help plants defend against degraded soil environments and enhance their ability to adapt to the environment by relieving environmental stress, releasing inorganic nutrients, and producing phytohormones or other mechanisms (Ali and Hasnain, 2007; Jansson and Hofmockel, 2020; Vries et al., 2020). As an important interaction factor with plants, rhizosphere microorganisms are essential for promoting plant growth and metabolic processes and enhancing soil fertility during phytoremediation (Li et al., 2015; Ulbrich et al., 2022). Some microorganisms can also decompose organic matter, transform soil nutrients, and participate in various soil biochemical reactions (Epelde et al., 2015; Sahu et al., 2017; Xue et al., 2018). Therefore, plants can cope with degraded soil environments by regulating root exudates to alter rhizosphere microorganism richness and diversity. Despite the well-documented importance of root exudates in phytoremediation, current research on root exudates mainly focuses on the idealized laboratory aspects of environmental pollutants, such as organic pollutants and heavy metals (Zhan et al., 2016; Du et al., 2020; Bian et al., 2021). Information on the changes in root exudates during the phytoremediation of degraded soil in the field is limited, and the dialog between plants and rhizosphere microbes remains unclear.

In this study, *Leymus chinensis* (Trin.) Tzvel. was chosen as the target plant. It has a well-developed root system and strong capacity for reproduction. *L. chinensis* reproduces asexually through powerful rhizomes and exhibits a strong resistance to abiotic stress and a strong potential for phytoremediation (Hu et al., 2022). We conducted a 3-year field experiment to investigate the effects of *L. chinensis* on the remediation of degraded soil fertility and structure. We aim to determine (1) how soil degradation affects the changes in soil bacteria, fungal communities and root exudate components and (2) how *L. chinensis* shapes microbial communities in response to degraded soil stress by regulating root exudates. We predicted that soil degradation would increase root exudate components. We also predicted that the changes in root exudate components would reshape soil microbial communities and enhance soil nutrients.

## Materials and methods

### Experimental site and setup

The research was conducted at the Degraded Soil Restoration and Treatment Demonstration Base (126°26′04″E and 45°57′87″N) at Northeast Agricultural University, Heilongjiang Province, China (Supplementary Figure S1). The area experiences a humid temperate continental monsoon climate with a mean annual average temperature of −5–4°C and a maximum average temperature of 23°C during the growing season. Eighty to ninety percent of the total precipitation occurs during the crop growing season, and the average annual precipitation is 500–650 mm. The predominant soil type in the area is Mollisol according to the United States Department of Agriculture (USDA) soil classification (Soil Survey Staff, 2003). We selected 3 representative degraded soils as experimental sites according to the land survey and the soil physical and chemical properties (Supplementary Table S1), including light (L), moderate (M) and high (H) degradation level soils. The L degraded soil was abandoned arable land. The M degraded sites were used as arable land over the same period, and the H degraded site experienced chronic overcultivation. The field experiment was established in July 2017, and *L. chinensis* was transplanted into the plot and cultivated evenly. The planting spacing was set as 30 cm, and the plot area was set as 5 m × 5 m. Each adjacent plot was an untreated plot as a buffer to minimize the crossover effect. Without fertilization and irrigation, the samples were harvested on September 3, 2020 after 3 years of complete natural recovery.

### Soil sample collection and determination

Rhizosphere soil was collected as described by Solá et al. (2019). In detail, *L. chinensis* were carefully uprooted with a shovel, and the soil around the roots was removed by gentle hand shaking. The soil tightly adhering to the root surface within 3 mm was collected in a sterile self-sealing bag, and the soil far from the root surface was considered the non-rhizosphere soil. Rhizosphere soil contains abundant root exudates, which makes its properties different from those of non-rhizosphere soil (Wang et al., 2022a). The three rhizosphere soils were marked as LR, MR, and HR, and the non-rhizosphere soils were marked as LN, MN, and HN. Samples were divided into two parts. One portion was naturally air-dried and passed through a 2-mm sieve to determine soil physical and chemical properties, and the remainder was stored at −80°C for further analysis. The air-dried soil sample was impregnated with an epoxy resin under vacuum, cut into 20 × 30 mm^2^ pieces, and adhered to the sample stage by conductive tape. Then, the samples were lapped with a silicon carbide paste and a diamond paste to reach a thickness of 32 μm. Finally, a 100–150 A metal film was coated on the sample surface by a plasma beam sputtering coating system (E-1010 HITACHI), and the morphology and structure of the soil aggregates were observed by scanning electron microscopy (SEM) (Hitachi S-4800 microscope; Allegretta et al., 2022). Soil pH and electrical conductivity (EC) were measured by a pH metre and EC metre (1:5 soil:water ratio, m:v), respectively. Total nitrogen (TN), ammonia nitrogen (AN), nitrate nitrogen (NN), total phosphorus (TP) and available phosphorus (AP) were measured using a continuous flow analyzer (SEAL Auto Analyzer 3). SOM was analyzed by the potassium dichromate method (Lin et al., 2022).

### 16S rRNA and its gene sequencing of soil microorganisms

Total DNA of rhizosphere soil and non-rhizosphere soil samples was extracted according to the manufacturer’s manual by a Power Soil DNA Isolation Kit (Mo Bio Laboratories, Inc., United States), and 1% agarose gel electrophoresis was used to detect quality and purity. The bacterial 16S rRNA gene was amplified using primers 338F (ACTCCTACGGGAGGCAGCAG) and 806R (GGACTACHVGGGTWTCTAAT) specific to the V3-V4 variable region. The fungal ITS rRNA gene was amplified using primers ITS1-F (CTTGGTTCATTTAGAGGAAGCTA) and ITS2 (TGCGTTCTTCATCGATGC) specific to the ITS1 region. The purified polymerase chain reaction (PCR) product was subsequently paired-end sequenced on the Illumina MiSeq platform (Allwegene Technologies Co., Ltd., Beijing, China). The high-quality sequences with ≥97% similarity were classified as the same operational taxonomic unit (OTU) and annotated by the Silva database and Unite database to classify the soil microbial communities into phenotypes. Soil microbial community diversity and richness information were analyzed by QIIME (v1.8.0).

### Root exudate collection and metabolite profiling

*Leymus chinensis* were collected randomly from each plot, rinsed with Milli-Q water carefully, and then disinfected with 2% Hg_2_Cl_2_. The root exudates were collected by the *L. chinensis* root system dipped in a dark conical flask containing 200 ml sterile Milli-Q water every 2 h. After multiple collections, the collected root secretion solution was combined until 1,000 ml of root exudate at each degradation level was obtained. The samples were filtered through a 0.45-μm filter, 40 ml of the samples was freeze-dried and extracted with 1,000 μl of extract (3:1 methanol:water ratio, V:V), and 10 μl of adonitol (0.5 mg mL^−1^) was added as an internal standard. The mixture was centrifuged at 12,000 rpm for 15 min at 4°C, and the supernatant was derivatized with methoxyamine hydrochloride (20 mg mL^−1^ in pyridine), BSTFA (1% TMCS, v/v) and FAMEs (in chloroform) for further analysis. Root exudates were ultimately separated by a DB-5MS capillary column (30 m × 250 μm × 0.25 μm, J&W Scientific, Folsom, CA, United States) and analyzed with gas chromatography time-of-flight mass spectrometry (GC-TOFMS). The metabolic markers were identified by the LECO-Fiehn Rtx5 database after filtering and normalizing the resulting data. The quality control samples for root exudate analysis were prepared by mixing aliquots of all samples.

### Statistical analysis

Root exudate analysis was performed with six biological replicates (*n* = 6), and the remaining experiments were performed in triplicate (*n* = 3). Data are presented as the mean ± standard errors and were analyzed by one-way analysis of variance (ANOVA). SPSS 19.0 and Origin Pro 9.0 software were used to analyze the significance of differences between groups, and *p* < 0.05 was considered statistically significant. Statistical tests were performed on the α diversity of the microbial data for each group, and differences between the groups were assessed. The R vegan package (version 3.3.2) was used to draw Venn diagrams showing the number of OTUs shared between samples, visually illustrating the overlap of OTUs between each sample. The top 10 phyla and genera with high abundance were filtered from the OTUs of bacteria and fungi, and the abundance composition heatmap of the top 10 phyla and genera in different treatments was plotted using R version 3.3.2. Principal coordinate analysis (PCoA) with a paired Bray–Curtis similarity matrix was performed by the OTU count statistics of 97% similarity between different samples. Linear discriminant analysis-effect size (LEfSe) analysis was used to determine the effect size of linear discriminant analysis (LDA), and the Wilcoxon rank sum test (α = 0.05) was used to identify species with abundance that differed significantly between categories. A cut-off of LDA values>3 was used to estimate the influence of species that differed significantly. The different root exudate metabolites were screened by variable importance in projection (VIP) scores and Student’s t test and identified using biochemical pathways with the Kyoto Encyclopedia of Genes and Genomes (KEGG) database. Correlations between bacteria, fungi and root exudate composition were calculated by Pearson correlation analysis. The R vegan package (version 3.3.2) was used to plot the correlation heatmap between bacteria, fungi and root exudate composition (*p* < 0.05 indicates significantly correlated, and *p* < 0.01 indicates strongly significantly correlated).

## Results

### Effects of *Leymus chinensis* root exudates on soil physicochemical properties

The physical and chemical properties of rhizosphere soil and non-rhizosphere soil in each treatment were measured to evaluate the soil remediation effects of root exudates of *L. chinensis*. The SOM, TN, AN, NN, TP, AP, pH and EC were significantly affected in every plot. The soil nutrient content was significantly improved by a 3-year phytoremediation, and the rhizosphere soil containing root exudates had higher nutrients than non-rhizosphere soils (Table 1). The SOM in LR, MR and HR was increased by 0.30-, 0.37- and 0.27-fold compared with LN, MN and HN, respectively. For the soil N content, TN, NH_4_^+^-N and NO_3_^−^-N were significantly increased by phytoremediation, among which root exudates increased TN by 2.37, 2.94, and 3.17%, respectively, compared with non-rhizosphere soil. Root exudates significantly increased the content of soluble N in the soil, with NH_4_^+^-N increasing by 40.52, 15.30, and 24.46% in LR, MR and HR, respectively. The increase in NO_3_^−^-N was higher than that in NH_4_^+^-N, increasing by 57.14, 47.93 and 70.03% in LR, MR and HR, respectively. The TP content increased slightly, but the AP significantly increased by 25.15, 32.60 and 36.46%, respectively. Notably, root exudates significantly decreased the soil pH by 1.43, 0.89 and 1.11% in LR, MR and HR, respectively.

**Table 1:** Changes of soil physical and chemical parameters in different plots.

Treatments	pH	EC (μS cm^−1^)	TN (%*10^−1^)	NO_3_^—^N (mg kg^−1^)	NH_4_^+^-N (mg kg^−1^)	TP (%*10^−2^)	AP (mg kg^−1^)	SOM (%)
LR	7.71 ± 0.03e	206.03 ± 3.66b	1.69 ± 0.03d	72.97 ± 2.19a	10.36 ± 0.16e	19.90 ± 0.04a	35.38 ± 0.94a	6.46 ± 0.16a
LN	7.82 ± 0.08d	173.23 ± 4.35a	1.65 ± 0.01d	43.40 ± 1.83C	4.44 ± 0.43c	18.04 ± 0.04b	28.27 ± 1.06c	4.96 ± 0.13c
MR	7.86 ± 0.03 cd	222.13 ± 5.78c	1.36 ± 0.01c	51.30 ± 3.22b	6.99 ± 0.66d	18.56 ± 0.06ab	32.66 ± 0.94b	6.11 ± 0.47a
MN	7.93 ± 0.06bc	203.27 ± 4.14b	1.32 ± 0.01bc	43.45 ± 0.50c	3.64 ± 0.14bc	17.45 ± 0.04b	24.63 ± 2.11c	4.47 ± 0.19d
HR	8.08 ± 0.01a	268.17 ± 4.07d	1.26 ± 0.02ab	48.97 ± 4.97b	3.47 ± 0.12b	15.72 ± 0.06c	29.08 ± 0.16d	5.60 ± 0.20b
HN	8.17 ± 0.02b	229.00 ± 2.64c	1.22 ± 0.01a	36.99 ± 0.05d	1.04 ± 0.18a	11.66 ± 0.16d	21.31 ± 1.55e	4.41 ± 0.14d

### Effects of *Leymus chinensis* root exudates on the soil structure

**Figure 1:**
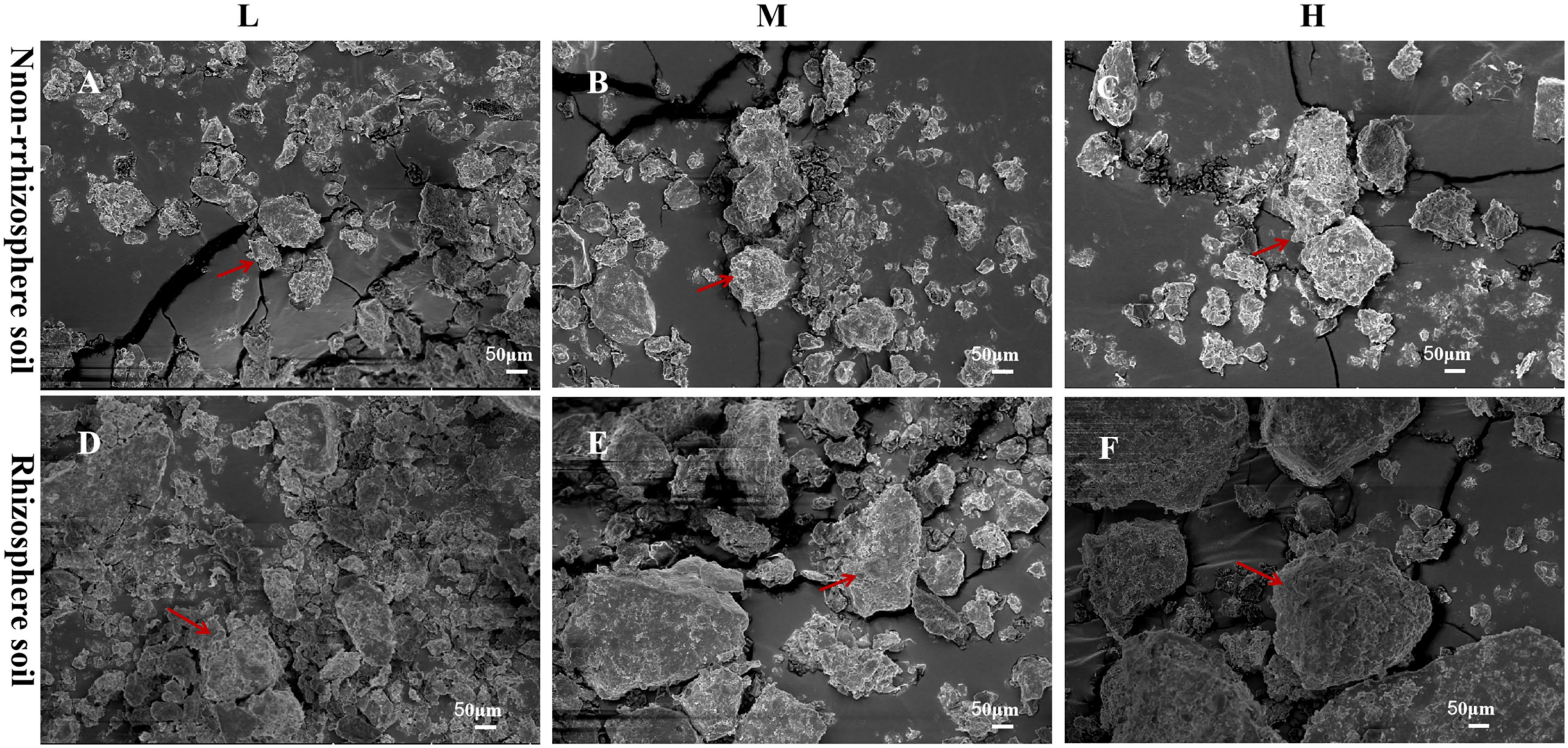
Scanning electron microscopy (SEM) images of the soil structure in each plot; **(A–C)** the rhizosphere soils in L, M, and H, respectively; **(D–F)** the non-rhizosphere soils.

The microstructures of the rhizosphere and non-rhizosphere soil samples for each treatment were imaged by SEM (Figure 1). The soil samples of each treatment contained soil aggregates. The SEM showed that the number of soil aggregates gradually decreased with increasing soil degradation level from the L, M and H groups, and the lowest number of soil aggregates was observed in the H group. Regarding aggregate size, the size gradually increased with the soil degradation level, and small and dense soil aggregates were noted in the L group. Root exudates from the same soil degradation level significantly altered the soil structure. The SEM displayed higher numbers of soil aggregates in the RL, RM and RH groups. Compared with NL, NM and NH, soil aggregates in the rhizosphere soil simultaneously showed larger particle sizes.

### Microbial community in *Leymus chinensis* planting soil

**Figure 2:**
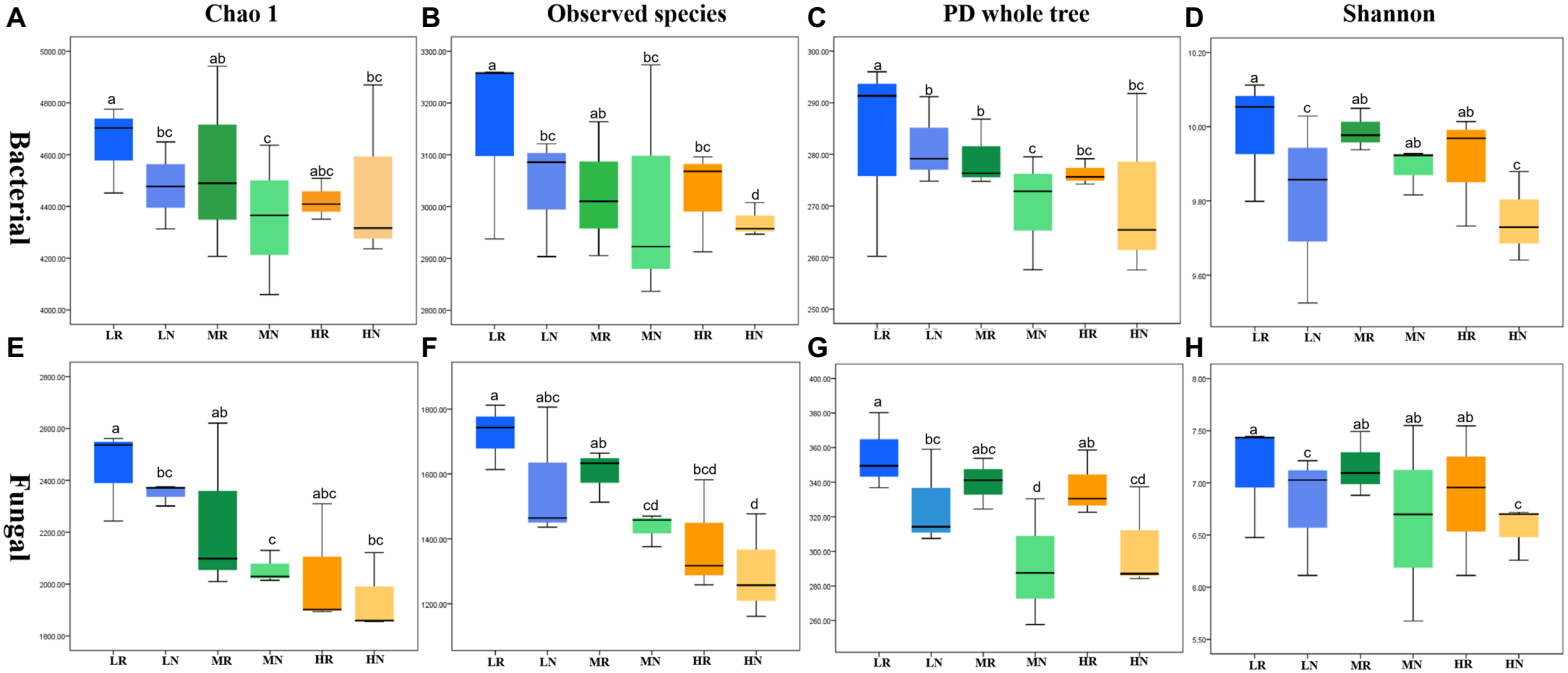
The α diversity in different soil samples. Bacteria **(A)** Chao1, **(B)** observed species, **(C)** PD whole tree, **(D)** Shannon index and fungal **(E)** Chao1, **(F)** observed species, **(G)** PD whole tree, **(H)** Shannon index.

The high-throughput Illumina saturation sequencing data showed that the rarefaction curve tended to be flat, indicating that the depth of sequencing data was reasonable (Supplementary Figures S2A,B). A total of 2,011,425 bacterial raw reads and 2,044,097 fungal raw reads were obtained from 18 soil samples. The bacterial and fungal data were clustered into 7,861 and 907 OTUs, respectively. In total, 2,782 bacterial and 907 fungal OTUs showed strong fitness, and each treatment contained specific microbiomes (Supplementary Figures S2C,D). The number of bacterial and fungal OTUs was higher in rhizosphere soils than in non-rhizosphere soils. The same trend was observed for the number of specific OTUs in both bacteria and fungi. The four indexes Chao1, observed species, PD whole tree and Shannon were used to compare species richness and diversity to examine differences in soil microbial community characteristics among treatments. There were significant differences in all indexes for each treatment. Among the bacterial communities, L exhibited the largest α diversity index followed by M and H, indicating that soil degradation reduced soil microbial diversity (Figures 2A–D). These indexes in rhizosphere soil showed that root exudates significantly restored some of the bacterial diversity and richness. Fungal communities showed similar trends to bacterial communities with root exudates showing effective effects on restoring soil microbial diversity (Figures 2E–H).

**Figure 3:**
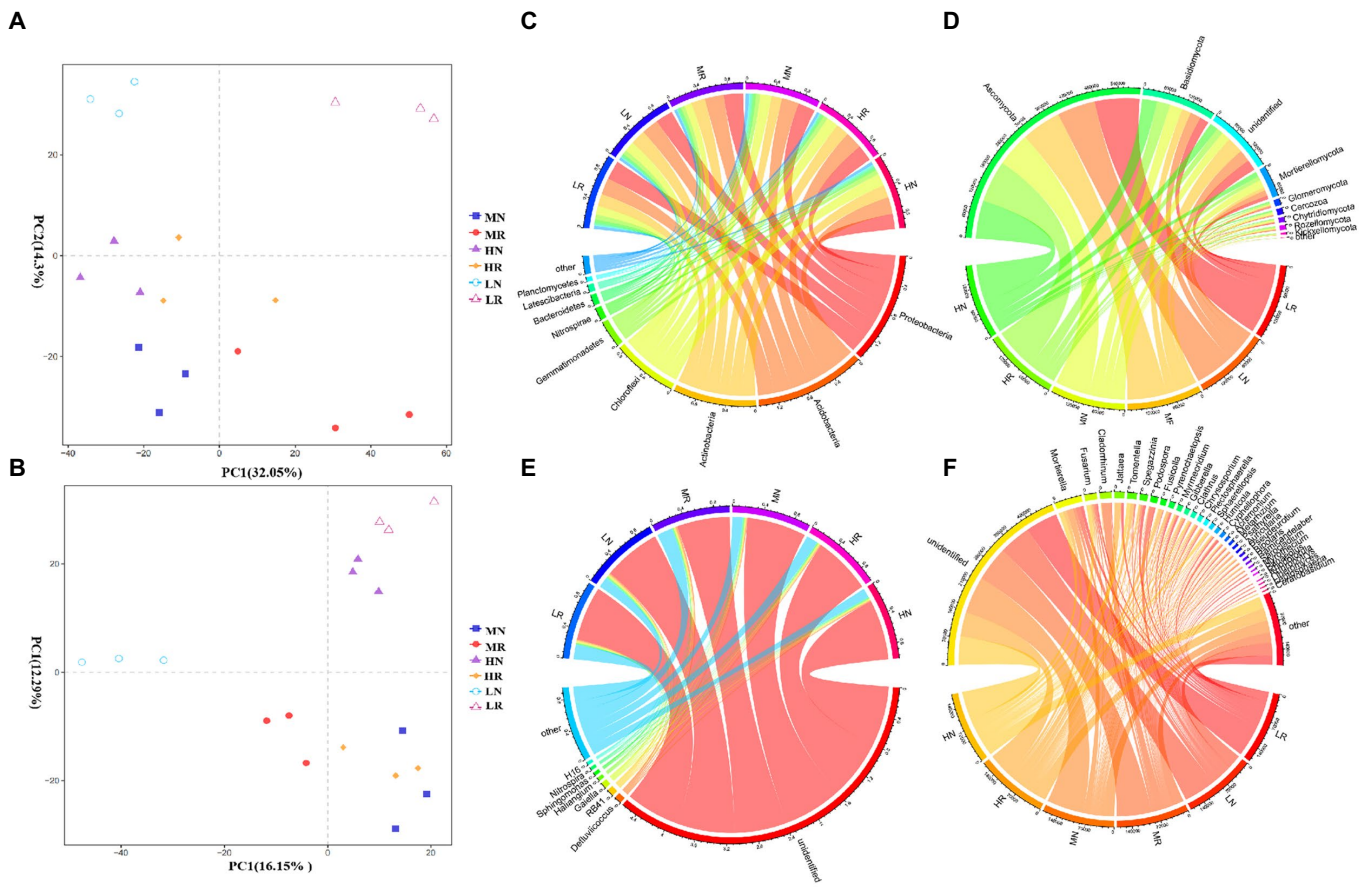
The PCoA diagram and relative abundances of dominant microorganisms in different treatments. **(A)** PCoA diagram of bacterial and **(B)** fungal microbial communities; **(C)** dominant bacterial and **(D)** dominant fungal species composition at the phylum level; **(E)** dominant bacterial and **(F)** dominant fungal species composition at the genus level.

PCoA revealed that PC1 had the greatest influence on species composition (32.05%), and PC2 explained 14.3% of the variance (Figure 3A). The same treatment groups were clustered together, indicating that all treatments exhibited good reproducibility. However, all rhizosphere soils were clearly separated from non-rhizosphere soils, indicating a significant difference among these treatments. At the phylum level, the dominant phyla of the LR, MR, and HR bacterial communities were *Acidobacteria*, *Proteobacteria*, *Actinobacteria*, *Chloroflexi* and *Gemmatimonadetes* (Figure 3C), and the relative abundances varied among treatments. Root exudates consistently enriched for *Acidobacteria*, *Proteobacteria*, and *Actinobacteria* and dispelled *Chloroflexi* and *Gemmatimonadetes*. The abundance of most bacterial taxa at the genus level changed significantly. Root exudates increased *RB41*, *Dedluviicoccus* and genera with a relative abundance <1% (rare taxa). LR attracted *Sphingomonas* and dispersed *H116*, and MR dispersed *Nitrospira* and *Haliangium* (Figure 3D). The fungal community PCoA showed that PC1 and PC2 explained 16.15 and 12.29% of the variation in species composition, respectively (Figure 3B). The rhizosphere and non-rhizosphere soil samples were clearly distinguished. Except for unidentified fungi, *Ascomycota* was the most abundant phylum in each treatment followed by *Basidiomycetes* and *Mortierella*, which became the dominant phyla (Figure 3E). Root exudates attracted more *Ascomycetes*, with the opposite trend noted for *Basidiomycota* and *Mortierella*. At the genus level, root exudates consistently dispelled *Mortierella* and enriched *Pyrenochaetosis*, *Cladosporium* and *Spegazzinia* (Figure 3F).

### Significantly enriched biomarker analysis

**Figure 4:**
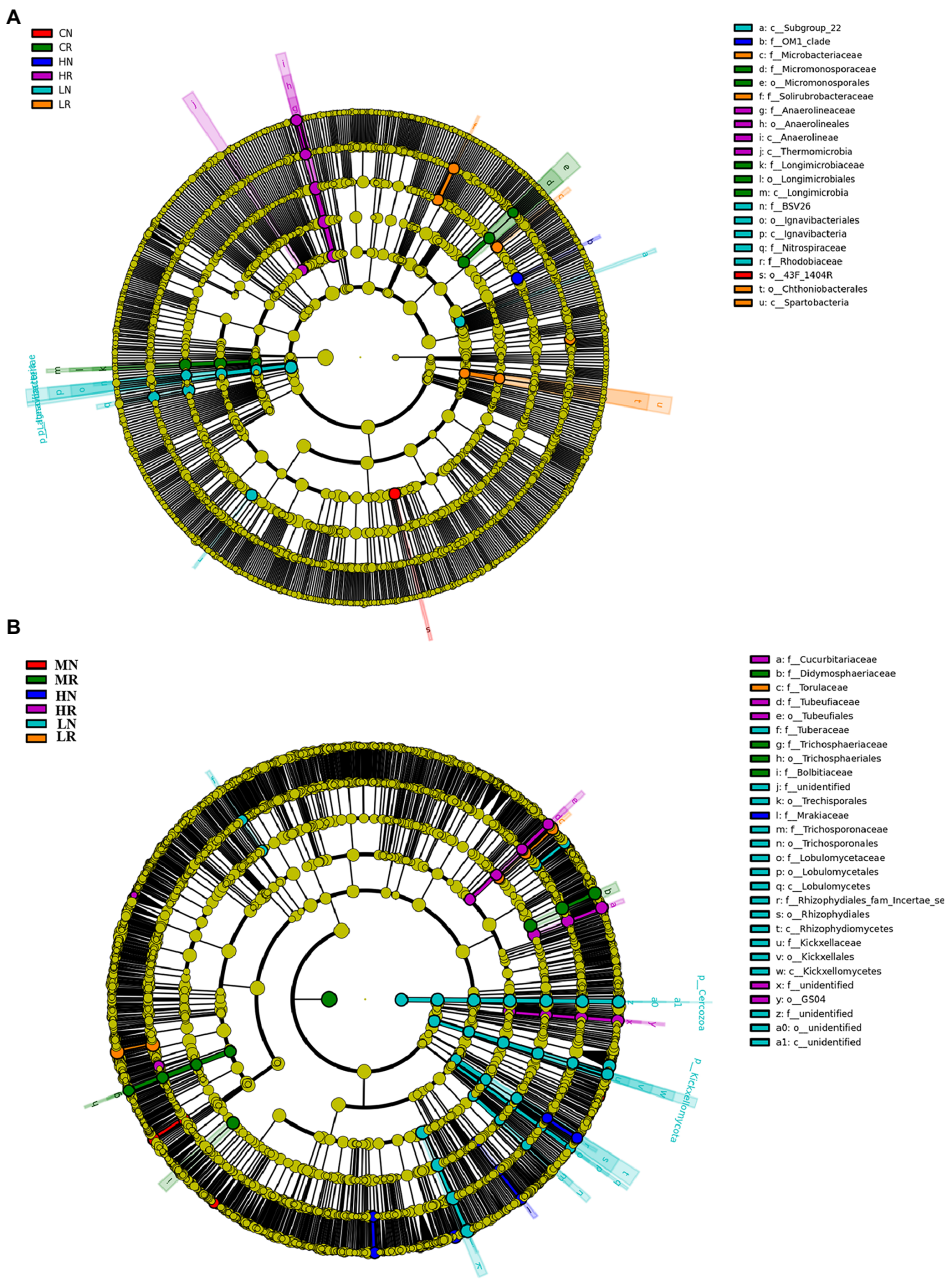
Species enriched biomarker diagram associated with each treatment. **(A)** Bacterial microbial community significantly enriched in each sample; **(B)** fungal microbial community significantly enriched in each sample. The size of each circle is proportional to the relative abundance of a community. Biomarkers were determined by LDA effect size (LEfSe) analysis, LDA scores >3, *p* < 0.05.

In total, 21 significant biomarkers were found in bacteria. A total of 6 significant biomarkers (1 order, 2 classes, and 3 families) were identified in LR, and *Lgnavibacteria* and *Lgnavibacteriales* were significantly enriched. LN had 4 significant biomarkers (1 order, 1 class, 2 families), and *Spartobacteria* and *Chthoniobacterales* were significantly enriched. Five significant biomarkers (1 order, 1 class, and 2 families) were identified in MR, which was significantly enriched in *Micromonosporaceae* and *Micromonosporales*. The most prominent order, *43F-1404R*, was a significant biomarker in MN. Four significant biomarkers (1 order, 2 classes, and 1 family) were identified in HR, and *Thermomicrobia* was significantly enriched. The *OM1 clade* was the only significant biomarker in HN (Figure 4A, histogram of the LDA scores is shown in Supplementary Figure S3A).

For the fungal microbial community, a total of 28 significant biomarkers were detected. In total, 17 significant biomarkers (6 orders, 4 classes, and 7 families) were identified in LR, which was significantly enriched in *Rhizophydiales* and *Rhizophydiomycetes*. LN was enriched in *Torulaceae*, which emerged as a significant biomarker. For the M treatment, 4 significant biomarkers (1 order and 3 families) were exclusively identified in MR, which was significantly enriched in *Didymosphaeriaceae* and *Bolbltoaceae*. Five (2 orders and 3 families) and 1 family biomarkers were significantly enriched in HR and HN, respectively. *Tubeufiaceae*, *Tubeufiales* and *Cucurbitariaceae* were significantly enriched in HR, and *Mrakiaceae* was enriched in HN (Figure 4B, histogram of the LDA scores is shown in Supplementary Figure S3B). The above results indicated that soil degradation affects microbial species composition, and root exudates improved the soil microbial species composition.

### Effects of degraded soils on root exudates of *Leymus chinensis*

**Figure 5:**
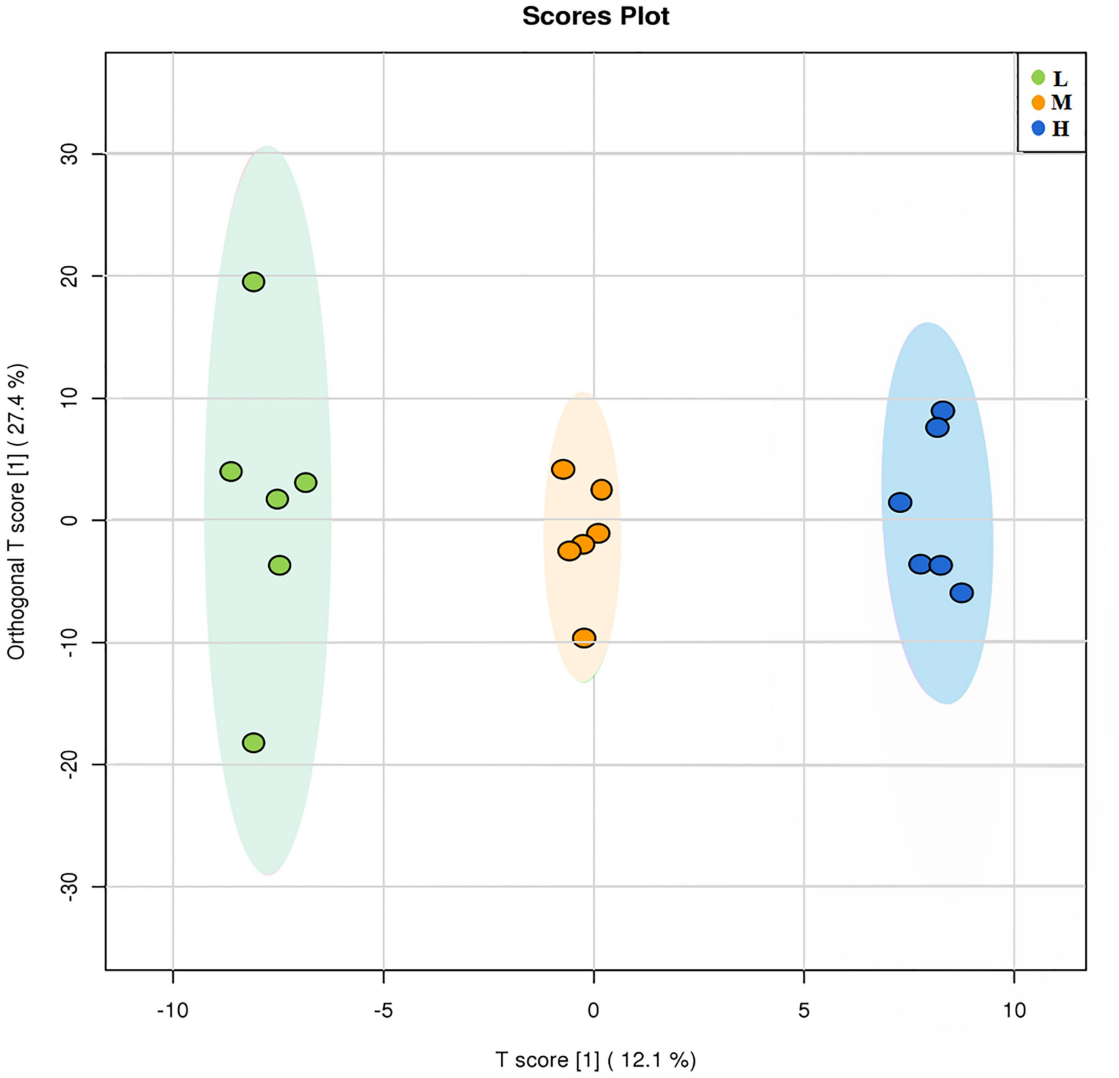
OPLS-DA of different degraded soil samples.

A total of 473 metabolites were detected in the GC-TOFMS chromatogram of root exudates of *L*. *chinensis*. OPLS-DA was applied to compare the changes in root exudates at different soil degradation levels (Figure 5). The OPLS-DA score plot results showed that each treatment was segregated along PC1, indicating that soil degradation significantly changed the components of root exudates. The abundance of metabolites changed significantly in response to soil degradation for 43 out of 473 compounds, as detected by Student’s t test (*p* ≤ 0.05) and VIP score (VIP > 1). These metabolites mainly involve organic acids, fatty acids, amino acids and other metabolites. The main metabolic changes were concentrated in nonpolar metabolites, including saturated and unsaturated long-chain fatty acids, such as palmitic acid, stearic acid, arachidic acid, and lauric acid. Some compounds with anti-abiotic stress properties were also altered by adaptation to degraded soil environments.

### Correlation of the soil microbiome and root exudates

**Figure 6:**
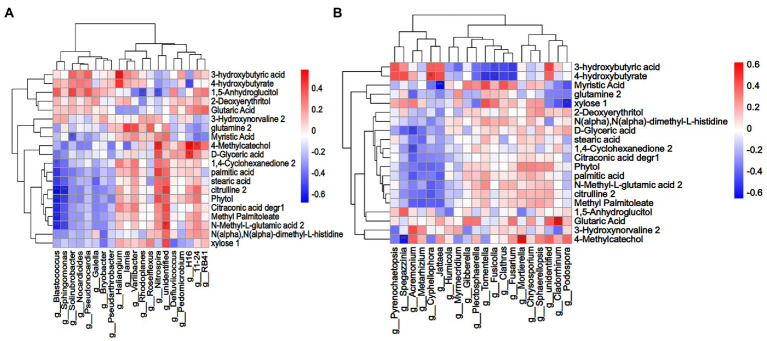
**(A)** Bacterial and **(B)** fungal Spearman correlation clustering heatmap between soil microorganisms (genus level) and differential root exudate components. Each column indicates a different microbial, and each row corresponds to the root exudate components. Red indicates a positive correlation, and blue indicates a negative correlation. A darker color indicates a greater correlation, and a lighter color indicates a lower correlation. **p < 0.01, *p < 0.05.

To explore the relationship between root exudates of *L*. *chinensis* and rhizosphere microorganisms, Spearman correlation analysis was performed between rhizosphere microorganisms with the top 20 relative abundances at the genus level and 43 differential root exudates (Figure 6). A total of 20 bacteria-related root exudate components were screened. The results revealed that in the bacterial microbial community, *Blastococcus* was strongly significantly negatively correlated with citrulline, phytol, citraconic acid degr, methyl palmitoleate and N-methyl-L-glutamic acid and significantly negatively correlated with 1,4-cyclohexanedione, palmitic acid and stearic acid. *Sphingomonas* was significantly negatively correlated with N-methyl-L-glutamic acid and phytol and strongly significantly negatively correlated with citrulline. Myristic acid was significantly negatively correlated with *Solirubrobacter*. *Haliangium* was significantly positively correlated with 3-hydroxybutyric acid, and *unidentified bacterium* was significantly positively with N-methyl-L-glutamic acid. 4-Methylcatechol was significantly positively correlated with *Nitrospira*, *H16* and *11–24*, and D-glyceric acid was significantly positively with *H16*. The relative abundance of *Blastococcus* in the LR, MR and HR treatments was 71.18, 74.12 and 63.80% lower than that in LN, MN and HN, respectively. *Sphingomonas* decreased by 41.46, 43.65 and 49.01% in the LR, MR and HR treatments, respectively. In LR, the relative abundance of *Haliangium* increased by 26.81% compared to LN. In addition, MR increased by 17.86% and HR increased by 36.63%. The relative abundance of *unidentified bacterium* increased by 12.12, 2.36 and 6.47% in LR, MR and HR, respectively. The relative abundance of *Nitrospira*, H16 and 11–24 also showed an increasing trend in each degradation level of rhizosphere soil. For fungi, the genera *Fusicolla* and *Fusarium* showed a significant negative correlation with 3-hydroxybutyric acid in root secretions. Notably, 4-hydroxybutyrate was significantly negatively correlated with the 4 genera *Tomentella*, *Fusicolla*, *Clathrus* and *Fusarium* but significantly positively correlated with *Cyphellophora*. Myristic acid was strongly significantly negatively correlated with *Cyphellophora*. Xylose was significantly negatively correlated with *Podospora*, and *Acremonium* was negatively correlated with D-glyceric acid and 1,4-cyclohexanedione. 4-Methylcatechol was strongly significantly negatively correlated with *Spegazzinia*, whereas the opposite was true for *Mortierella*. Glutaric acid was strongly significantly positively correlated with *Cladorrhinum*. The relative abundance of *Fusicolla* was 29.50, 55.68 and 71.03% reduced in LR, MR and HR soils, respectively, compared with non-rhizosphere soils, and the relative abundance of *Fusarium* was 12.20, 6.56 and 6.73% lower, respectively. The same trend was observed for the 4 genera *Tomentella*, *Fusicolla*, *Clathrus* and *Fusarium*. In contrast, the opposite trend was observed for *Cyphellophora*, where the relative abundance increased by 72.20, 93.10 and 83.67% in LR, MR and HR soils, respectively. *Podospora* decreased by 3.11, 6.71, and 13.18%, and *Acremonium* decreased by 35.69, 15.91, and 54.98% in each degraded level rhizosphere soil, respectively. The relative abundance of *Cladorrhinum* in LR, MR and HR increased by 95.96, 72.14 and 51.03%, respectively.

## Discussion

Phytoremediation is an environmentally friendly and efficient soil remediation technology that can fix carbon by photosynthesis and reach the soil by rhizodeposition of belowground or litter materials above-ground litter materials to ensure long-term sustainability of soil quality, but the effects on living root systems, such as root exudates, are less known (Pausch and Kuzyakov, 2018). In this research, we investigated the effects of root exudates of *L*. *chinensis* on the physicochemical properties and structure of soil. Root exudates improved soil fertility and increased the content of insoluble nutrients. Root exudates also increased soil aggregate numbers and improved soil structure. Soil degradation can reduce the composition and diversity of the soil microbial community. This process is not conducive to soil ecological function, but soil function can be recovered after the release of root exudates. In addition, *L. chinensis* could adapt to degraded soil environments by modulating its own metabolic system to alter root exudate components, and the root exudate components could attract potentially beneficial microorganisms.

### Effects of root exudates on soil properties and structure

We hypothesized that root exudates of *L. chinensis* would increase soil fertility given that root exudates can fix carbon by photosynthesis in the soil to ensure long-term soil quality sustainability. In our study, phytoremediation significantly increased SOM in the L, M and H treatment groups, especially in the rhizosphere soil containing root exudates. This difference can be attributed to the continuous input of root exudates providing more SOM to the soil. The compilation of the results of different studies (quantitatively examining the effect of root carbon input on organic carbon) has shown that the main source of SOC content is root-derived C inputs (Rasse et al., 2005; Jackson et al., 2017). Consistent with our results, Shen et al. (2020) found that soil degradation increased the root exudation rate, which increased the SOC content. In addition, the macromolecular polysaccharides produced by root exudates of *L. chinensis* exhibit strong adhesion to soil particles and promote the formation of aggregates, which can facilitate the stabilization and sequestration of SOC (Huang et al., 2018; Kamran et al., 2021). Therefore, the increase in soil aggregates also promotes the SOM content, which is one reason why the fertility of the RL, RM and RH soils was greater than that of the non-rhizosphere soils. pH is one of the most important critical factors in soils. The cation–anion exchange equilibrium, redox reactions and organic acids released from root exudates can affect the rhizosphere pH (Wen et al., 2018). In our experiments, root exudates of *L. chinensis* contained a large number of amino acids and low molecular weight organic acids, such as citric acid, oxalic acid, malic acid, and succinic acid. These compounds possess a large amount of H^+^, which reduced the rhizosphere soil pH and acidified the soil (Table 1). These low molecular weight organic acids can also chelate inorganic ions to increase the availability of insoluble nutrients, which is also an important strategy for plants to increase nutrient uptake (Zhu et al., 2018). This mechanism might explain why NH_4_^+^-N, NO_3_^−^-N and AP in the RL, RM and RH groups were presente at higher levels than those in non-rhizosphere soil. In addition, soil aggregates can provide a good production space for soil microorganisms (Lin et al., 2018). The diversity and structure of soil microbial communities are crucial to the performance of soil functions and the ecological environment (Fierer, 2017; He et al., 2022). These findings support our initial hypothesis that the presence of root exudates improved soil fertility, increased the content of insoluble nutrients, increased soil aggregate numbers and improved soil structure.

### Effects of root exudates on the bacterial and fungal diversity and community structure in different degraded soils

In our study, the diversity and composition of bacterial and fungal microbial communities in the L, M and H rhizospheres and non-rhizosphere soil were determined by 16S rRNA and ITS gene high-throughput sequencing. The sequencing results showed that soil degradation disrupted the composition and diversity of soil microbial communities, which is detrimental to soil ecological function. OTU results showed that the rhizosphere soil contained more specific OTUs in bacteria and fungi, demonstrating the recruitment of more new microorganisms into rhizosphere soil to resist soil stress (Lin et al., 2019). The α-diversity results confirmed that *L. chinensis* increased the diversity and richness of rhizosphere soil bacterial and fungal communities. This finding is noted because root exudates promote an increase in soil nutrients and SOM, enabling rapid microbial community growth and higher colony numbers than those noted in non-rhizosphere soils. Signaling substances in root exudates can attract specific soil microorganisms and alter the microbial community structure. PCoA results also demonstrated that root exudates significantly altered the soil microbial community structure. These results suggest that phytoremediation is an effective method to improve soil microbial abundance and diversity. Overall, our results showed that the cultivation of *L. chinensis* ameliorated the soil degradation-induced reduction in the number and diversity of microbes (Figure 5), and the effect of root exudate production in the rhizosphere soil was more positive, effectively resisting the adverse soil environment. Qu et al. (2020) reported that a more diverse microbial community structure provided better tolerance to abiotic and biotic stresses.

Soil functions are strongly linked to the composition of microbial communities. In the current study, *Proteobacteria* and *Actinomycetes* were dominant genera and accounted for more than half of the relative abundance, and compared with non-rhizosphere soils, the relative abundance significantly increased in rhizosphere soil. These genera are important plant-associated bacteria that can provide better tolerance against biotic and abiotic stresses (Palaniyandi et al., 2013). *Actinomycetes* breakdown plant litter by producing extracellular enzymes, and some genera can produce regulatory substances (such as IAA) to promote plant growth and improve soil nutrients (Ali et al., 2019). The increase in soil nutrient levels could attract the colonization of *Ascomycetes* as the niche was more suitable for the *L. chinensis* rhizosphere environment with high soil nutrients, and *Ascomycetes* serves as the main indicator phylum of soil fungal communities. This phylum has significant metabolic flexibility, which makes it applicable in various biotechnologies, including the degradation of soil pollutants and the production of biofuels (Johnson, 2013). It is also widely used in plant pathogens, animal pathogens, and biotechnology industries (Hagee et al., 2020). For fungi, root exudates enhanced the competitiveness of *Ascomycetes*, which could help overcome the competition between *Basidiomycetes* and *unidentified bacterium* and make the relative abundance of *Ascomycetes* in rhizosphere soils higher than that noted in non-rhizosphere soils. Root exudates also recruited more *Humicola*, which can be used as a biological control agent to reduce the incidence of *Phytophthora capsicum* and cabbage black spot (Yang et al., 2014). Notably, the abundance of beneficial microorganisms, such as *Haliangium*, *Sphingomonas*, *Nitrospira*, *Mortierella*, *and Cladorrhinum*, which have positive effects on plant growth and soil health, was significantly enriched in the rhizosphere soil (Luo et al., 2020; Li et al., 2020a; Yin et al., 2022; Wang et al., 2022b). A similar result was found in *Arabidopsis thaliana* after infection with pathogens, demonstrating plants can recruit potentially beneficial microbes to improve resistance to adverse external environments (Yuan et al., 2018). Overall, these results indicate that root exudates may recruit some potentially beneficial microbes to resist soil degradation stress and that the ecological functions and biogeochemical processes of soil ecosystems are also changing.

### Effects of root exudate composition changes on the soil microbiome in different degraded soils

Plants can alter the components of root exudates, which may shape specific rhizosphere microorganisms and improve the root zone soil environment. Our findings suggest that the abundance of several potentially beneficial microorganisms was significantly increased in the rhizosphere soil, and *L. chinensis* might recruit microorganisms to the rhizosphere by root exudates. To further test this hypothesis, we conducted correlation analysis to determine the relationship between the abundance of rhizosphere microorganisms and root exudates. Correlation analysis results indicated that the same root exudate components may have positive or negative regulatory effects on different bacteria and fungi, and the same microorganisms may have different nutritional preferences. Correlation analysis of *Arabidopsis thaliana* root exudates and the rhizosphere microbiome also yielded similar results (Liu et al., 2020). Plant roots can recruit growth-promoting bacteria by secreting organic acids. Qu et al. (2021) reported that hexanoic acid and β-alanine exhibited recruitment capacity to the potential beneficial bacteria *Rhizobiaceae* and *Burkholderiaceae*. Malic acid can induce systemic resistance in plants by attracting *Paenibacillus polymyxa* and *Bacillus amyloliquefaciens* (Martins et al., 2018). We observed that glutaric acid and N-methyl-L-glutamic acid potentially serve as nutrient sources for *Cladorrhinum* and *unidentified bacterium*. Similarly, 3-hydroxybutyric acid might serve as a nutrient source for *Haliangium* and also dispelled the pathogenic bacteria *Fusicolla* and *Fusarium*, improving the plant living environment. In addition to organic acids, we observed a positive correlation between 4-methylcatechol and the potentially beneficial *Nitrospira* and *Mortierella*, suggesting that it could serve as a nutrient source for these two microorganisms. However, 4-methylcatechol was negatively correlated with *Spegazzinia*, indicating that 4-methylcatechol significantly reduced their ability to compete. *L. chinensis* altered the secretion of glutamic acid, 3-hydroxybutyric acid, and 4-methylcatechol in response to soil degradation and thus recruited *Haliangium*, *Nitrospira*, and *Mortierella* to the rhizosphere, implying that the recruitment of rhizosphere microorganisms with potential soil remediation functions may be regulated by soil degradation-induced root exudates. These results suggested that plants can communicate with soil microbes through root exudates, which alter microbial competition, recruit potentially beneficial rhizosphere soil microbes, reshape soil microbial communities and resist biotic and abiotic stresses.

## Conclusion

This study verified that *L. chinensis* phytoremediation enhanced the degraded soil nutrient content, improved the soil structure, and maintained soil sustainability. *L. chinensis* remedied the decrease in soil microbial species diversity caused by soil degradation and improved the soil microbial flora. *L. chinensis* recruited some potentially beneficial rhizosphere microorganisms, and soil degradation altered the levels of organic acids, fatty acids and amino acids in root exudates. Notably, the correlation of bacterial and fungal abundances with root exudates indicated that glutaric acid, 3-hydroxybutyric acid and 4-methylcatechol recruited *Haliangium*, *Nitrospira* and *Mortierella* to the rhizosphere to disperse the harmful microorganisms *Fusicolla* and *Fusarium*. This finding suggests that *L. chinensis* cultivation is an effective method for improving soil quality and restoring soil microbial ecosystems.

## Data availability statement

The data presented in the study are deposited in the NCBI repository, accession number PRJNA902702.

## Author contributions

YL and LM performed the data analyzes and wrote the manuscript. QW, BL, and PZ contributed significantly to analysis and manuscript preparation. SS performed the experiment. GC contributed to the conception of the study. All authors contributed to the article and approved the submitted version.

## Funding

This work was financially supported by the National Natural Science Foundation of China (32201470), the Heilongjiang Provincial Natural Science Foundation of China (LH2020C021), the National Key R&D Program of China (2016YFC0500607), the Heilongjiang Postdoctoral Fund to pursue scientific research (LBH-Z20003), and the Young Talents Project of Northeast Agricultural University (19QC21). Each of the funding bodies granted the funds based on a research proposal. The bodies had no influence on the experimental design, data analysis and interpretation, or writing of the manuscript.

## Conflict of interest

The authors declare that the research was conducted in the absence of any commercial or financial relationships that could be construed as a potential conflict of interest.

## Publisher’s note

All claims expressed in this article are solely those of the authors and do not necessarily represent those of their affiliated organizations, or those of the publisher, the editors and the reviewers. Any product that may be evaluated in this article, or claim that may be made by its manufacturer, is not guaranteed or endorsed by the publisher.

## Supplementary material

The Supplementary material for this article can be found online at: https://www.frontiersin.org/articles/10.3389/fmicb.2022.951838/full#supplementary-material
