# Supplementary material for: Leymus chinensis resists degraded soil stress by modulating root exudate components to attract beneficial microorganisms
Source: Front Microbiol. 2022 Dec 9;13:951838. doi: 10.3389/fmicb.2022.951838 (PMC9780673; doi:10.3389/fmicb.2022.951838)
Supplement: Supplementary file 1 [file Data_Sheet_1.docx]

Table S1 Initial physical and chemical properties of three representative degraded soils.

| Treatments | Land use patterns | pH | EC | TN | NO_3_^−^-N | NH_4_^+^-N | TP | AP | SOM |
| --- | --- | --- | --- | --- | --- | --- | --- | --- | --- |
|  |  |  | (μS cm^−1^) | (%*10^-1^) | (mg kg^−1^） | (mg kg^−1^) | (%*10^-2^) | (mg kg^−1^) | (%) |
| L | abandoned arable land | 8.01 | 356.33 | 0.74 | 27.57 | 3.9 | 14.28 | 17.64 | 3.14 |
| M | arable land | 8.14 | 382.33 | 0.61 | 30.81 | 3.13 | 12.47 | 28.49 | 1.2 |
| H | experienced chronic over-cultivation | 8.41 | 429 | 0.47 | 36.17 | 1.02 | 11.43 | 19.63 | 0.82 |

| No. | Name | No. | Name | No. | Name |
| --- | --- | --- | --- | --- | --- |
| 1 | Citraconic acid 3 | 2 | 3-Hydroxynorvaline 2 | 3 | fumaric acid |
| 4 | 3-hydroxybutyric acid | 5 | Glutaric Acid | 6 | 4-hydroxybutyrate |
| 7 | 4-Methylcatechol | 8 | Phenyl beta-D-glucopyranoside | 9 | Sophorose 2 |
| 10 | lauric acid | 11 | 4-hydroxy-3-methoxybenzoic acid | 12 | beta-Glutamic acid 2 |
| 13 | glutamine 2 | 14 | Myristic Acid | 15 | 2-Monopalmitin |
| 16 | heptadecanoic acid | 17 | Methyl Phosphate | 18 | N(alpha),N(alpha)-dimethyl-L-histidine |
| 19 | Diglycerol 1 | 20 | 2-deoxy-D-glucose 2 | 21 | 6-phosphogluconic acid |
| 22 | Maleimide | 23 | xylose 1 | 24 | 4-Hydroxybenzoic acid |
| 25 | piceatannol 2 | 26 | Arachidic acid | 27 | Fluorene |
| 28 | N-Methyl-L-glutamic acid 2 | 29 | N(epsilon)-Trimethyllysine | 30 | oleic acid |
| 31 | Phytol | 32 | 1,3-Cyclohexanedione 1 | 33 | Citraconic acid degr1 |
| 34 | palmitic acid | 35 | Methyl Palmitoleate | 36 | stearic acid |
| 37 | 1,4-Cyclohexanedione 2 | 38 | citrulline 2 | 39 | 2-Deoxyerythritol |
| 40 | 1,5-Anhydroglucitol | 41 | 4-Hydroxy-6-methyl-2-pyrone | 42 | D-Glyceric acid |
| 43 | ferulic acid |  |  |  |  |

Table S2 Differential components of *L. chinensis* root exudates under three different degraded soils.
