# Supplementary figures and images for: Leymus chinensis resists degraded soil stress by modulating root exudate components to attract beneficial microorganisms
Source: Front Microbiol. 2022 Dec 9;13:951838. doi: 10.3389/fmicb.2022.951838 (PMC9780673; doi:10.3389/fmicb.2022.951838)

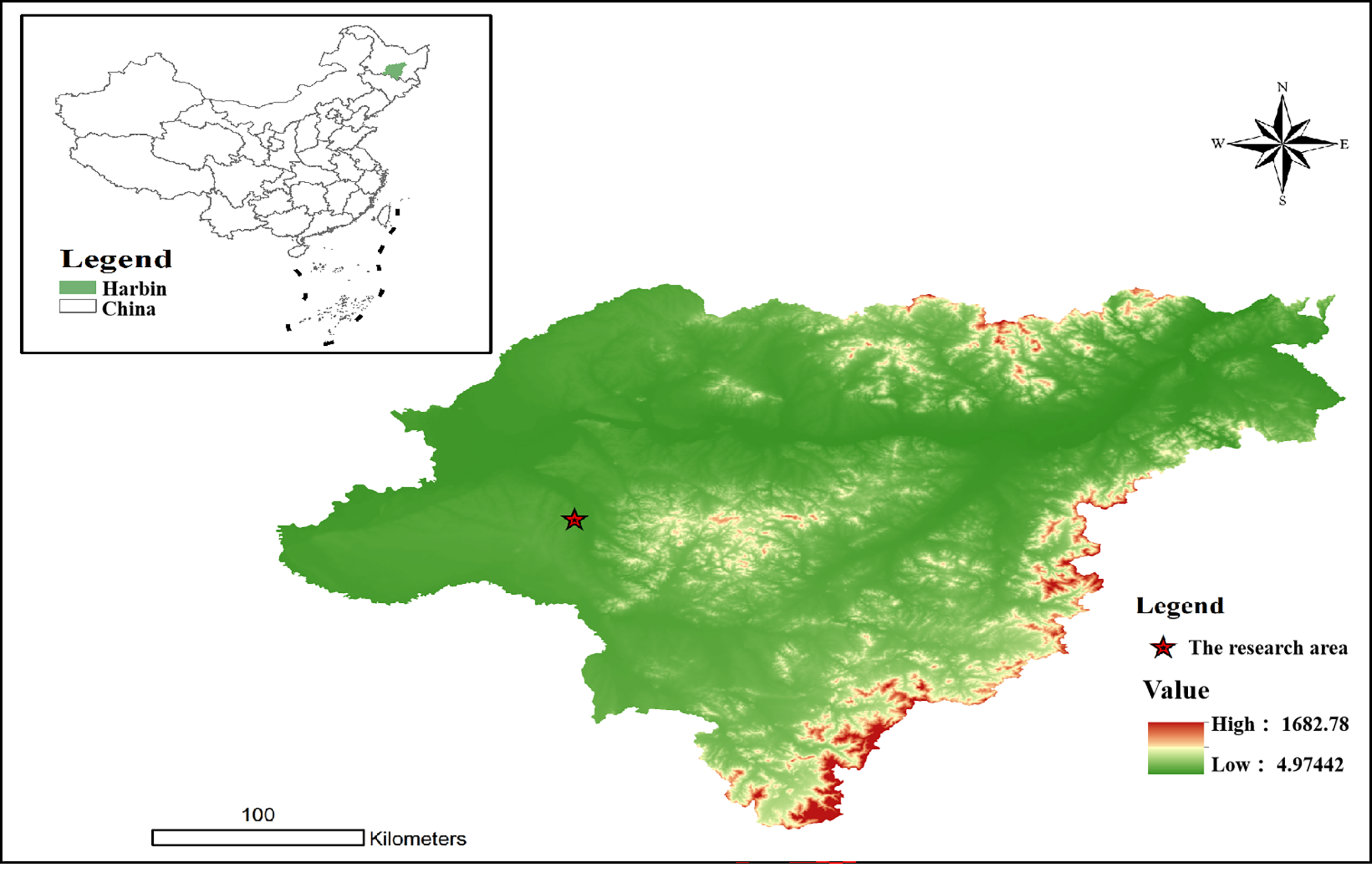

Supplement: Supplementary file 2 [file Image_1.TIF]

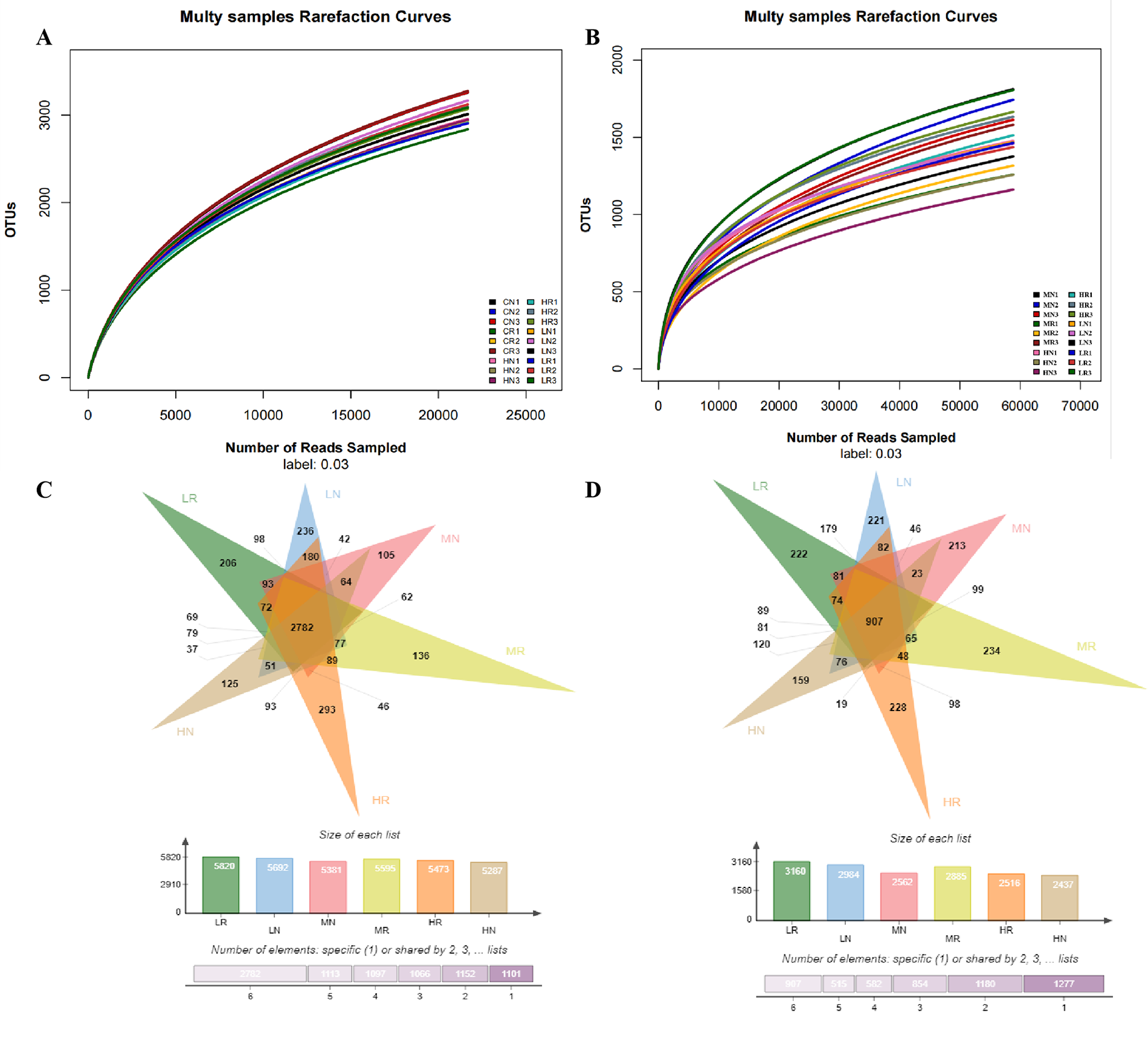

Supplement: Supplementary file 3 [file Image_2.TIF]

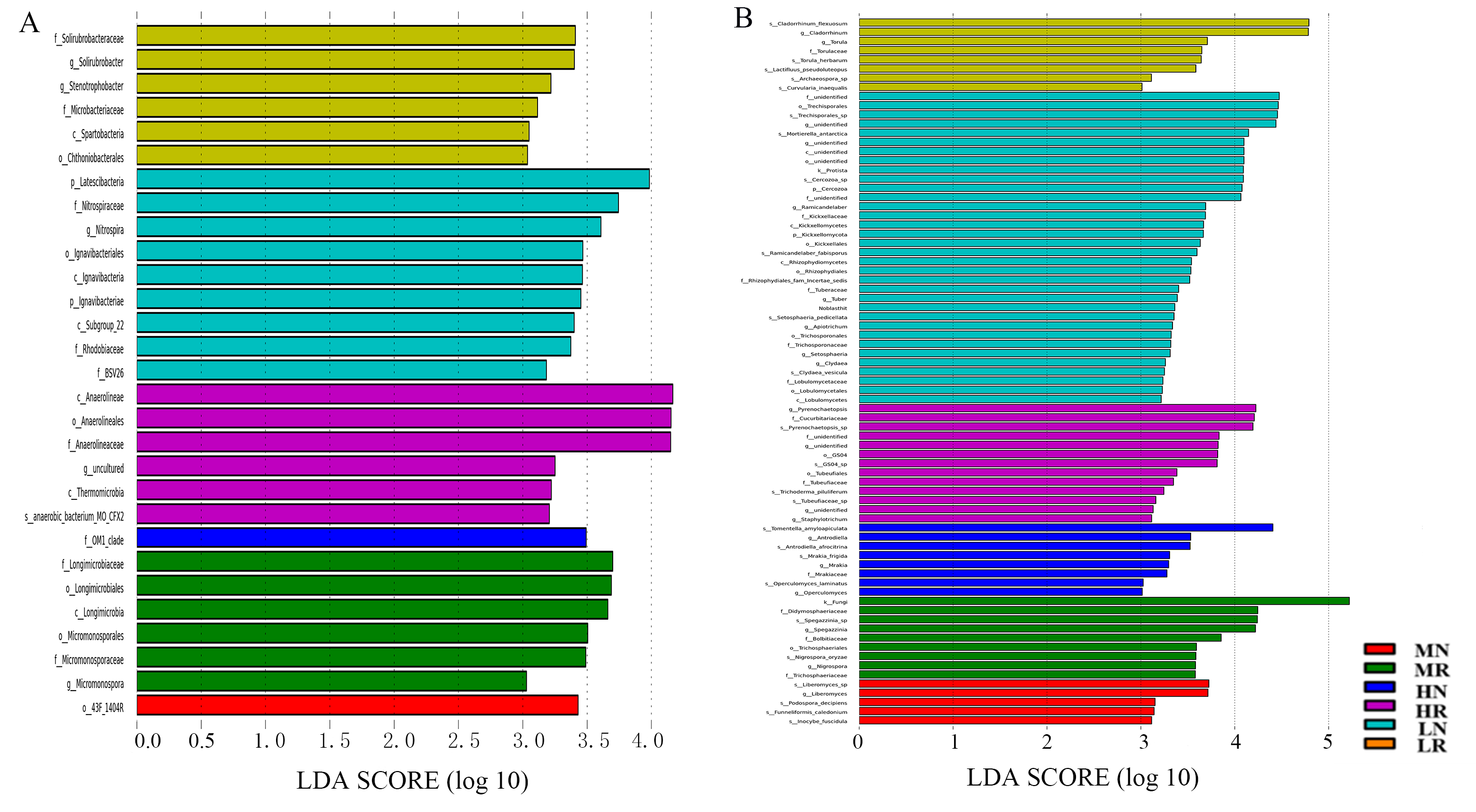

Supplement: Supplementary file 4 [file Image_3.TIF]
